# Inflammation of the Neck of the Bladder

**Published:** 1888-04

**Authors:** Jno. Thad. Johnson

**Affiliations:** Atlanta, Ga.


THE
### Southern Medical Record.

A MONTHLY JOURNAL OF PRACTICAL MEDICINE.

☞ All Communications must be addressed to R. C. WORD, M. D., Managing Editor.

"*Quicquid Præcipies Esto Brevis.*"

VOL. XVIII. ATLANTA, GA., APRIL, 1888. No. 4.

### ORIGINAL AND SELECTED ARTICLES.

### INFLAMMATION OF THE NECK OF THE BLADDER.

By JNO. THAD. JOHNSON, M. D.

A most obstinate and disagreeable complication, or sequel, of gonorrhœa is an inflammation of that part of the deep urethra and the immediately adjacent surface of the bladder, which, together, are ordinarily known as the neck of the bladder.

This inflammation varies in intensity. But even in its slightest form, in that degree in which the inflammatory processes are not pronounced, the symptoms are so annoying, so distressing, as to call for relief.

I call special attention to this disease, not alone because of its importance and its frequency, but because I am sure it is often overlooked; or, more correctly, because the symptoms are misinterpreted, and the real seat and nature of the lesion not recognized.

Probably I can best present the subject by giving the history of a patient who recently came under my care. This is but a type of a large class:

A young man had, with the assistance of experienced friends, conducted the treatment of his gonorrhœa for several weeks after its appearance. At this period, the symptoms became more alarming, or more distressing. The scalding of the urethra during micturition had been less severe for sometime than it was at the outset. But now there were frequent calls for evacuating the bladder, even when the viscus was almost entirely empty. Indeed, the desire was almost constant. The tenesmus attending and following each act was considerable. The burning pain low in the pubic region was quite marked. There was some little mucus passed with the urine. These, it is true, are but the symptoms of inflammation of the bladder in a mild form. Yet, it had not once occurred to the patient's mind that the disease had passed beyond the range of his little weapon—the ordinary urethral syringe.

He had consulted a physician who evidently had misinterpreted the symptoms, for he only recommended a change of the urethral injection.

It is evident that in this case the parts below the compressor had become involved, by continuity of tissue, and that the ordinary injection failed to reach the diseased surface. Two or three days' treatment, properly directed, relieved the urgency of the case. The pain, tenesmus, and frequency of urination were all greatly modified; but all remained, in a lessened degree, for a long time. The patient continued his avocation, but was always conscious of an uneasiness in this region, and had frequent calls to pass his urine; these calls proving to have been wholly unnecessary so far as indicated by the amount of fluid expelled.

Patients so affected are especially apt to suffer an exacerbation of all the symptoms when subjected to undue exercise, prolonged standing, walking, horse-back riding, and imprudence in drinking. They are generally sensitive to changes of weather.

This extension of inflammation, as before said, is often entirely overlooked, or goes unrecognized. In such instances treatment of the anterior urethra is persistently adhered to, and much surprise is felt that no beneficial result is obtained. This slight inflammation of the neck goes on indefinitely. The patient occasionally has reason to hope he is at last rid of it; but the effect of any of the etiological conditions above mentioned rudely undeceive him.

The mucus discharged, of course, varies with the varying intensity of the inflammation as well as with the greater or less extent of surface of the mucous membrane involved. It is generally distributed through the expelled urine, floating therein as a flocculent cloud. A portion may be passed separately at the very outset of micturition. More often still do we find the quantity greatest just at the close of the act. This is especially true when the inflammation is most active. The mucous then is not only more abundant, but is also more tenacious, and renders the tenesmus more violent.

The mucus is observed in another form, which sometimes greatly alarms the patient. At the commencement of urination, threads or little ropes are detected as they emerge from the meatus. The patient imagines that all manner of evil is thus shown to exist. This form of mucus, I believe, is more apt to arise from a chronic inflammation of the urethra just in front of the compressor than from the part under our special consideration. At any rate, however, it may be co-existent with the inflamed neck.

There are yet other circumstances under which this mucus may present itself in a manner alarming to the patient. Months have passed, he is pursuaded that his long-standing gonorrhœa has at last exhausted itself; all the severe symptoms have disappeared or been greatly mitigated, or, perhaps, were mild from the outset; or its true character, or location, possibly, was never suspected. The patient accidentally discovers this slight cloud of mucus floating in the urine after it has been passed in a vessel. His fears are magnified by the writings which have fallen into his hands. and which were written for this very purpose. He precipitately flies for comfort, in all likelihood, to the p ace where he doesn't get it—to some charlatan who has bated his trap so carefully with this very fear moving morsel, for this very kind of victim. I have known physicians to allow themselves honestly misled by this symptom. I drew more especial attention to this in a paper entitled "Some Disorders, Miscalled Spermatorrhœa," which I presented several years since to the Georgia Medical Association.

I have known the inflammation extended from the urethra to the vesical neck by means of a catheter, which the patient passed into the bladder with a view of making more effective treatment of his troublesome gonorrhœa in its earlier stage.

It can scarcely be necessary. after saying so much of the symptoms, to enter largely into the question of diagnosis. I have urged the necessity of proper appreciation of the symptoms that mark the passage of the inflammation beyond the compressor urethra muscle, in order that our treatment, or our methods, may be adapted to this further extension.

We should bear in mind the possibility of the involvement of the prostate gland. If this be acutely inflamed, one might mistake it for a general cystitis; but not probably for the condition we are considering. There would be present a burning pain with some tenesmus; some mucus might be thrown off. But the pain is in the perinæum, extending toward the rectum. The finger passed into the lower bowel, would at once recognize an acute prostatitis.

As a sequela of urethritis, an inflammation of the prostatic ducts, with the peculiar conditions of the discharge of the prostatic mucus, is by no means infrequent. This is generally known as prostatorrhœa. The mere calling of attention to it should be sufficient to suggest the means for a differential diagnosis. This is also treated of in the paper already referred to.

As in the last instance, so in acute (general) cystitis, and in catarrh of the bladder (chronic cystitis) should the mere suggestion of their names be sufficient to avoid such a mistake in diagnosis.

The symptoms described as indicating an inflammation of the neck may attend a calculus in any part of the tract, or disease of the kidneys, or even tuberculosis or other disease of the prostate. The history of the case, with the general make-up of the symptoms, will not likely leave cause for doubt in diagnosis.

With the inflamed neck, there also frequently is still present the disease in the anterior urethra. Here, of course, we have the discharge of mucus, or muco-pus, flowing from the urethra independent of urination; otherwise, it is observed only on such occasions.

We should bear in mind that the prostatic urethra is a portion, a large portion, of what is known as the neck of the bladder. We, therefore, must expect what we may term an interchange of symptoms between the diseased neck and the diseased prostate. The two may co-exist.

When the inflammation of the neck is severe, there often will appear a small quantity of blood with the muco-pus, especially at the close of micturition, when the tenesmus is greatest. The microscope may also show spermatozoa under the same circumstances This severity of symptoms, however, in a typical case of the diseases now treated of, is either altogether absent, or of short duration.

The sound or catheter will afford further light for our information. The natural sensitiveness of the deep urethra is here increased. A momentary spasm of the compressor muscle may be expected, and, if present, may be overcome by a persistent, gen le pressure with a blunt pointed sound.

It is natural to expect that what of burning pain may be present in these cases is felt more in the perinæum than in general cystitis. In the latter lesion, it is more marked in the pubic region. Pain of a reflex neurotic character may be complained of in almost any of the nerves of this region of the body. They are not peculiar to this catarrh of the neck. There is not infrequently present that general condition formerly called hypochondria, or hysteria, now described as a general neurosis, or, perhaps, as a neurasthenia. But however it may be named, it is alike troublesome to patient and physician.

The prognosis in this form of vesical catarrh depends greatly upon the stage in which treatment may be commenced, and the means adopted for its cure. Even at the best we may not be too sanguine of a speedy and perfect cure. In the many cases in which the location of the trouble is not properly made out, and the treatment adapted only to the anterior urethra is kept up, the patient must worry with the disease—now better, again worse—for an indefinite period.

This condition is often treated by the use of the sound. This I regard as not a good practice, however well it may be adapted to a gleet of the urethra proper. I have frequently known patients who had been subject, or had subjected themselves, to the frequent introduction of the sound for a long time without any improvement whatever. In most of such cases it is possible to effect a cure by discontinuing the sound and resorting to other measures. The good effect of treatment by the sound, in cases rightly selected, cannot be questioned; but like many good things it has been carried too far in more senses than one. Patients who are intrusted with an instrument often develop what I term the bougie habit, that amounts almost to a monomania.

At the outset of treatment, we must make careful inquiry, and lay down careful instructions, as to the patient's habits. In no disease is it more essential that these be correct. We can accomplish little if the patient be intemperate. His dress demands supervision; the bladder is sensitive to weather changes, or to exposure by the patient. Harmful exercise must be forbidden.

We generally cannot refrain, in treating this affection, from exhibiting remedies by the stomach. Their sometimes good effect is not denied; but there is an inglorious uncertainty about it that is not so attractive as the glorious uncertainty of the law is said to be in the eyes of the legal fraternity.

[TO BE CONTINUED.]

---

**Pleuritis.**—In a discussion on pleuritis in Marion County Medical Society, Dr. Ferguson said (*Indiana Medical Journal*):

In the treatment of acute pleuritis, thoracentesis is not done as frequently as it should be, and the result is that many cases are badly cured and subsequently die of empyema, or remain permanent invalids.

